# Supplementary material for: Reduced ambiguity and improved interpretability of bacterial genome-wide associations using gene-cluster-centric k-mers
Source: Microb Genom. 2023 Nov 7;9(11):001129. doi: 10.1099/mgen.0.001129 (PMC10711318; doi:10.1099/mgen.0.001129)
Supplement: Supplementary material 1 [file mgen-9-1129-s001.pdf]

Supplementary Material

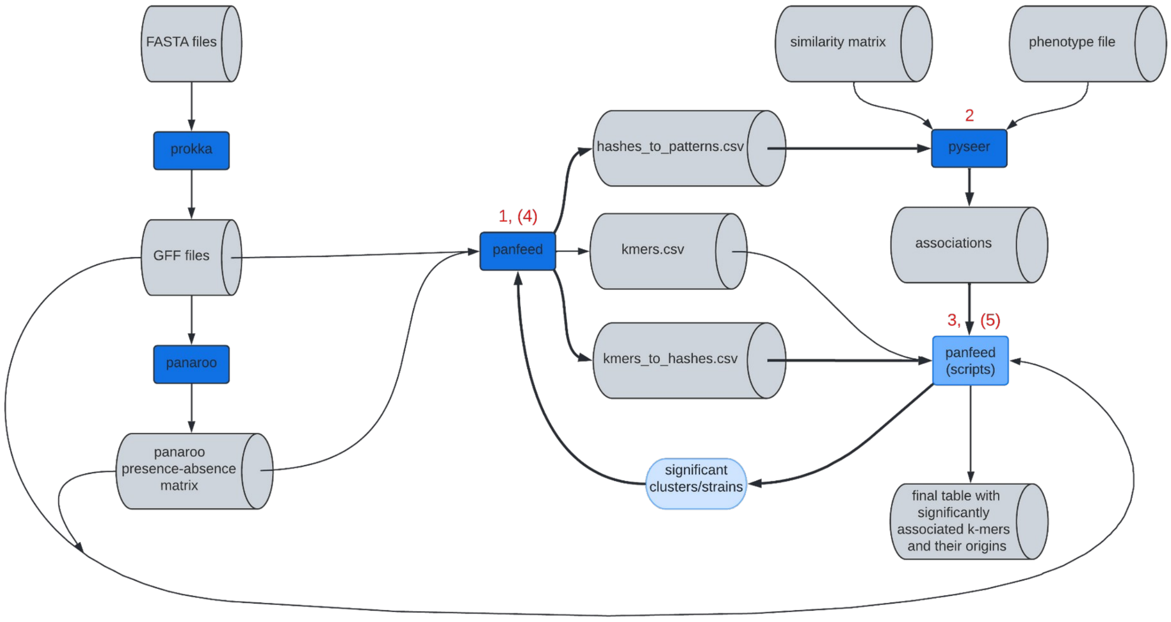

Supplementary Figure 1. Workflow for a GWAS that employs panfeed as k-mer generator. Bold arrows mark steps that can be repeated to reduce both the memory footprint as well as resulting disk space being used by the written files. Red numbers indicate the step at which the specific program or script is being used. Steps in parentheses indicate optional steps for the two-pass approach that reduces time consumption and disk usage.

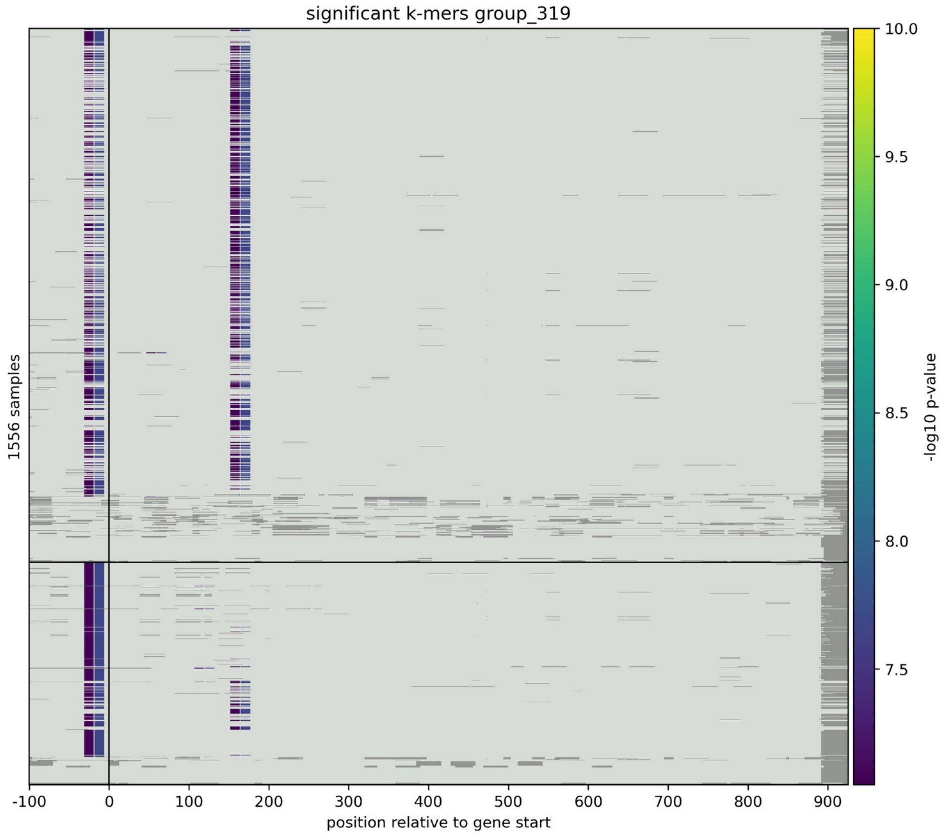

Supplementary Figure 2. Unshifted -log10 p-values of significantly associated k-mers in gene cluster group_319 (*fHbp*) as found by panfeed in the *N. meningitidis* data set. The figure only displays nucleotide positions up to base pair 925 to exclude irrelevant information, even though some genes reach a length beyond that point.

Clusters with significantly associated unitigs	Clusters with significantly associated k-mers
group_11364group_18381<br>group_19470<br>group_2782<br>group_5515<br>group_5516<br>group_6526<br>group_7266<br>group_7980intA_4~~~intS_2~~~intS_3~~~intA~~~intA_2~~~intA_3~~~intS_4~~~intA_1~~~intA_5~~~intS_5~~~intS_1~~~intS<br>intA_7~~~intS_3~~~intA_6~~~intA_3~~~intA_1~~~intS_1~~~intA_5~~~intS_2~~~intA_4~~~intS_4<br>intS_2~~~intS_4~~~intS_1~~~intA_5~~~intA_7~~~intA_1~~~intS_3~~~intA_4~~~intA_6~~~intA_3~~~intS_6~~~intS<br>intS_3~~~intS_4~~~intA_2~~~intA_1~~~intS_1~~~intA_3~~~intA_7~~~intA_4~~~intA_6<br>intS~~~intS_2<br>papC_4~~~papC_5~~~papC_2~~~papC_3~~~papC_1<br>papD_2~~~papD_3<br>papG<br>papG_1~~~papG<br>papG~~~papG_2<br>papH~~~papH_2<br>papK	group_5515<br>group_7980<br>papD_2~~~papD_3

Supplementary Table 1. Gene clusters containing significantly associated unitigs/k-mers.
